# Unraveling and characterization of novel T3SS effectors in *Edwardsiella piscicida*

**DOI:** 10.1128/msphere.00346-23

**Published:** 2023-08-29

**Authors:** Xiao Jian Liao, Tian Tian He, Lu Yi Liu, Xiu Long Jiang, Shan Shan Sun, Yu Hang Deng, Li Qiang Zhang, Hai Xia Xie, Pin Nie

**Affiliations:** 1 State Key Laboratory of Freshwater Ecology and Biotechnology, Institute of Hydrobiology, Chinese Academy of Sciences, Wuhan, China; 2 College of Advanced Agricultural Sciences, University of Chinese Academy of Sciences, Beijing, China; 3 Fisheries Research Institute, Wuhan Academy of Agricultural Sciences, Wuhan, China; 4 Laboratory for Marine Biology and Biotechnology, Pilot National Laboratory for Marine Science and Technology, Qingdao, China; 5 School of Marine Science and Engineering, Qingdao Agricultural University, Qingdao, China; U.S. Food and Drug Administration, Silver Spring, Maryland, USA

**Keywords:** comparative proteomics, secretome, T3SS effectors, *Edwardsiella piscicida*

## Abstract

Type III secretion system (T3SS) facilitates survival and replication of *Edwardsiella piscicida in vivo*. Identifying novel T3SS effectors and elucidating their functions are critical in understanding the pathogenesis of *E. piscicida. E. piscicida* T3SS effector EseG and EseJ was highly secreted when T3SS gatekeeper-containing protein complex EsaB-EsaL-EsaM was disrupted by EsaB deficiency. Based on this observation, concentrated secretomes of Δ*esaB* strain and Δ*esaB*Δ*esaN* strain were purified by loading them into SDS-PAGE gel for a short electrophoresis to remove impurities prior to the in-the gel digestion and mass spectrometry. Four reported T3SS effectors and two novel T3SS effector candidates EseQ (ETAE_2009) and Trx2 (ETAE_0559) were unraveled by quantitative comparison of the identified peptides. EseQ and Trx2 were revealed to be secreted and translocated in a T3SS-dependent manner through CyaA-based translocation assay and immunofluorescent staining, demonstrating that EseQ and Trx2 are the novel T3SS effectors of *E. piscicida*. Trx2 was found to suppress macrophage apoptosis as revealed by TUNEL staining and cleaved caspase-3 of infected J774A.1 monolayers. Moreover, Trx2 has been shown to inhibit the p65 phosphorylation and p65 translocation into the nucleus, thus blocking the NF-κB pathway. Furthermore, depletion of Trx2 slightly but significantly attenuates *E. piscicida* virulence in a fish infection model. Taken together, an efficient method was established in unraveling T3SS effectors in *E. piscicida*, and Trx2, one of the novel T3SS effectors identified in this study, was demonstrated to suppress apoptosis and block NF- κB pathway during *E. piscicida* infection.

**IMPORTANCE:**

*Edwardsiella piscicida* is an intracellular bacterial pathogen that causes intestinal inflammation and hemorrhagic sepsis in fish and human. Virulence depends on the *Edwardsiella* type III secretion system (T3SS). Identifying the bacterial effector proteins secreted by T3SS and defining their role is key to understanding *Edwardsiella* pathogenesis. EsaB depletion disrupts the T3SS gatekeeper-containing protein complex, resulting in increased secretion of T3SS effectors EseG and EseJ. EseQ and Trx2 were shown to be the novel T3SS effectors of *E. piscicida* by a secretome comparison between ∆*esaB* strain and ∆*esaB*∆*esaN* strain (T3SS mutant), together with CyaA-based translocation assay. In addition, Trx2 has been shown to suppress macrophage apoptosis and block the NF-κB pathway. Together, this work expands the known repertoire of T3SS effectors and sheds light on the pathogenic mechanism of *E. piscicida*.

## Introduction

*Edwardsiella piscicida*, previously known as *Edwardsiella tarda*, is a Gram-negative intracellular pathogen recognized as the causative agent of hemorrhagic septicemia in fish, and it is also an etiological agent of gastrointestinal and extraintestinal infections in humans (1 – 3). Central to the pathogenesis of *Edwardsiella* is the type III secretion system (T3SS) (4). Disruption of the T3SS increases its LD_50_ by 10 times (5). Effectors translocated via *E. piscicida* T3SS have been shown to be involved in evading host immune surveillance, promoting intracellular survival and replication, and facilitating colonization (6 – 9).

Bacterial pathogens inject T3SS effector proteins into host to establish a hospitable intracellular niche (10). The core components of T3SS are conserved by different bacteria; however, their T3SS effector repertoire shows remarkable strain specificity, resulting in distinct disease manifestations of individual pathogen. Over 32 T3SS effectors have been reported in *Salmonella*, 23 reported in *Shigella*, 8 in *Yersinia*, and more than 300 type IV secretion system effectors in *Legionella* (11 – 14). Based on the genes that are signiﬁcantly regulated when *E. piscicida* replicates inside macrophage, 20 novel translocation-positive T3SS effector candidates were screened (15). To date, only four *E. piscicida* T3SS effectors have been functionally characterized. Among them, EseG triggers microtubule destabilization (16); EseJ suppresses adhesion and invasion of *E. piscicida* and inhibits host apoptosis through negatively regulating type 1 fimbria (5, 8, 9); EseK inhibits mitogen-activated protein kinase phosphorylation and promotes bacterial colonization in zebrafish (7); EseH, an enzyme that belongs to the family of phosphothreonine lyase, inhibits phosphorylation of ERK1/2, p38α, and JNK MAPK pathways in host cells (6). In the genus of *Edwardsiella*, there is another pathogen*—Edwardsiella ictaluri*, which mainly infects catfish and zebrafish, and its invasion activates extracellular signal-regulated kinases 1 and 2 (ERK1/2) early in infection, which are subsequently inactivated by its T3SS effector EseN through dephosphorylation, resulting in increased proliferation (17 – 19). It is interesting to discover and characterize new T3SS effector proteins to elucidate the pathogenic mechanism of *Edwardsiella*.

Mass spectrometry (MS)-based proteomics is a highly sensitive and quantitative tool for examining protein mixtures. When properly controlled, proteomic profiling of bacterial secretome helps expand the effector repertoire of bacterial pathogens, e.g., in *Salmonella*, enteropathogenic *Escherichia coli*, *Citrobacter rodentium*, and *Bacillus cereus* (20 – 26). The protein complex EsaB-EsaL-EsaM in *E. piscicida* is homologous to SpiC-SsaL-SsaM in *Salmonella*, which is the T3SS gatekeeper-containing protein complex (27, 28). The secretion of T3SS effectors is suppressed by SpiC in *Salmonella* (29). EsaB in *E. piscicida* is the homolog of SpiC in *Salmonella* (28). In this study, concentrated secretomes of the Δ*esaB* strain and Δ*esaB*Δ*esaN* strain (T3SS mutant) were pre-purified before subjecting them to mass spectra. Through quantitative and comparative proteomics analysis and translocation assay, EseQ and Trx2 were identified as the novel T3SS effectors, and the role played by Trx2 was investigated.

## RESULTS

### Rationale for strains used for quantitative and comparative secretome

**Fig 1:**
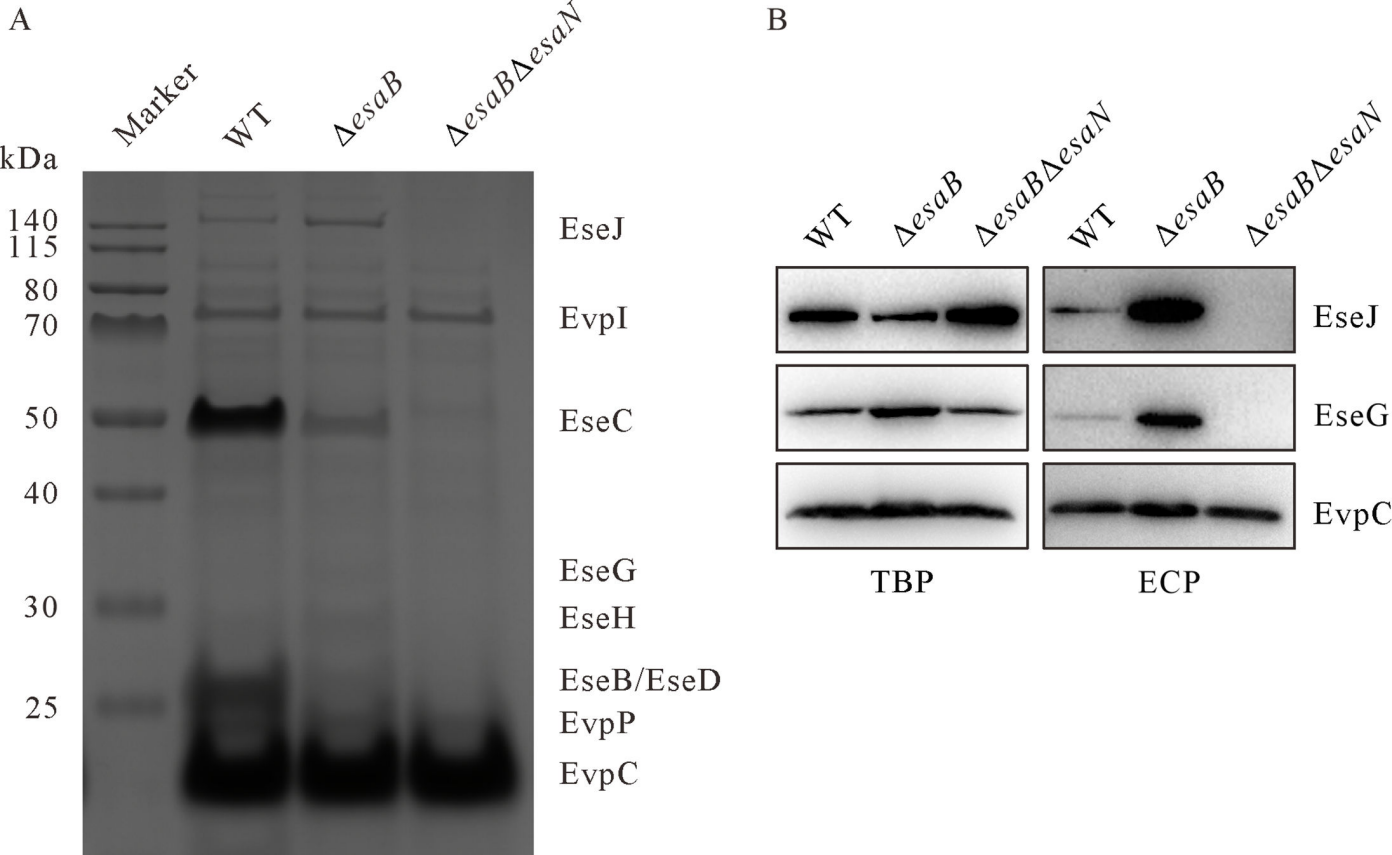
Secretion profiles of *E. piscicida* strains. The extracellular proteins and total bacterial pellets (TBPs) from similar amounts of bacteria grown in DMEM were sampled. (**A)** Secretion profiles of *E. piscicida* wild-type (WT) strain, Δ*esaB* strain, and Δ*esaB*Δ*esaN* strain. ECPs from similar amounts of bacteria grown in DMEM were concentrated before being separated by SDS-PAGE gel and stained with Coomassie blue. T3SS effector EseJ, EseG, and EseH; T3SS translocon EseC, EseB, and EseD; and T6SS protein EvpP, EvpI, and EvpC are indicated. (**B)** The immunoblotting for secretion of T3SS effector EseJ, EseG from *E. piscicida* wild-type strain, Δ*esaB* strain, and Δ*esaB*Δ*esaN* strain. EvpC, a protein secreted by T6SS was included to indicate a similar amount of protein loading per lane.

More than 20 T3SS effector candidates have been discovered over the years from *E. piscicida* by different approaches (15, 16, 30). Nevertheless, a comprehensive catalog of its T3SS effectors is lacking, complicating a better understanding of the pathogenesis of *E. piscicida*. Depletion of EsaB disrupts the T3SS gatekeeper-containing protein complex EsaB-EsaL-EsaM and disrupts the tightly controlled secretion of T3SS effectors at neutral pH in Dulbecco’s modified Eagle medium (DMEM) (28). Based on this, the extracellular proteins of the isogenic Δ*esaB* strain of *E. piscicida* were examined, with the Δ*esaB*Δ*esaN* strain as the control. EsaN is the T3SS ATPase that energizes the secretion and translocation of T3SS proteins (16). As expected, the T3SS effectors EseJ, EseG, and EseH [confirmed by Matrix-Assisted Laser Desorption / Ionization Time of Flight Mass Spectrometry (MALDI-TOF MS)] were highly secreted as their bands could be observed on SDS-PAGE gel from Δ*esaB* strain when compared to its parent WT strain or the isogenic Δ*esaB*Δ*esaN* strain (Fig. 1A). In parallel, extracellular proteins (ECPs) of Δ*esaB* strain or Δ*esaB*Δ*esaN* strain were probed with anti-EseG and anti-EseJ antibodies. Highly elevated EseG or EseJ was probed in ECPs of Δ*esaB* strain compared to those of the WT strain; neither EseG nor EseJ secretion could be detected from Δ*esaB*Δ*esaN* strain (Fig. 1B). EvpC, a protein secreted by type VI secretion system (T6SS), was included to indicate a similar amount of protein loading per lane (31). This indicates that by comparing the secretome of Δ*esaB* strain and Δ*esaB*Δ*esaN* strain, novel effector candidates could be unraveled.

### Eliminating low molecular weight impurities from secretomes

**Fig 2:**
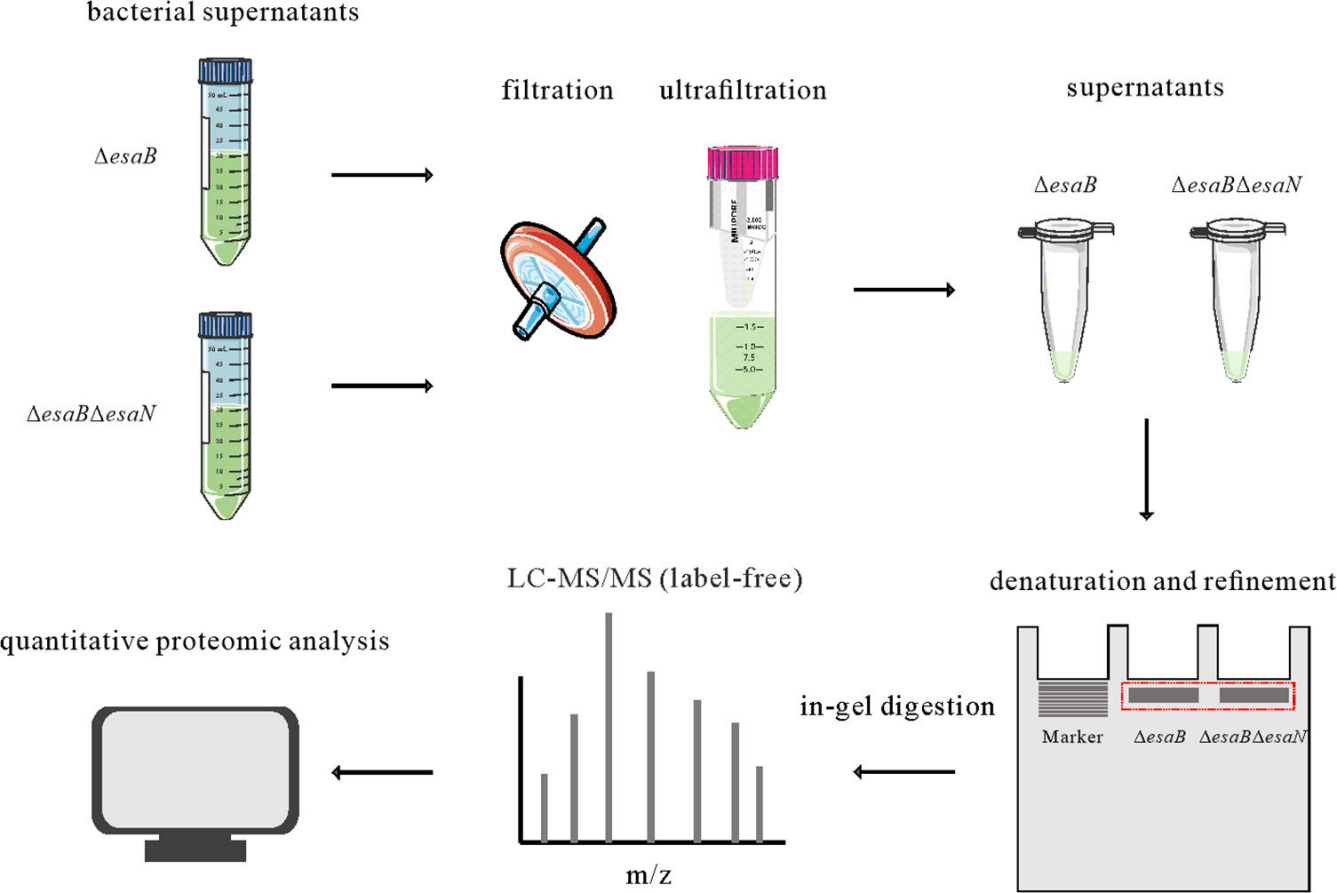
Optimized schematic overview of quantitative and comparative secretome of Δ*esaB* strain and Δ*esaB*Δ*esaN* strain. Briefly, Δ*esaB* strain and Δ*esaB*Δ*esaN* strain grown in DMEM were pelleted, culture supernatants were filtered and concentrated through centrifugal filter devices with a molecular weight cut-off of 3.0 kDa. ECPs obtained were denatured and loaded into SDS-PAGE gel and run at constant voltage of 70 V for 17 minutes to remove the low molecular weight impurity. After the protein sample entered the isolation gel, the gel was fixed, stained with Coomassie blue, and routinely destained before banding in-gel digestion prior to mass spectrometric LC-MS/MS analysis. Effector candidates were fished out by quantitative comparison on proteins obtained from Δ*esaB* strain and Δ*esaB*Δ*esaN* strain.

Culture supernatants of Δ*esaB* strain and Δ*esaB*Δ*esaN* strain were filtered and concentrated using Amicon Ultra-15 centrifugal filter device with 3.0 kDa molecular weight cut-off (Millipore). The supernatants acquired were loaded into 12% SDS-PAGE gel to run at 70 V for 17 minutes before gels were fixed, stained with Coomassie blue, and routinely destained. The protein loaded gels were cut for in-gel digestion and LC-MS/MS. With this step, the protein samples were pre-cleaned. On one hand, low molecular weight impurities from DMEM components, such as D-glucose, HEPES, amino acids, vitamins, inorganic salts, etc. are to be eliminated; on the other hand, proteins in gels have been denatured and further concentrated. The resulting mass spectra were searched against the genome of *E. piscicida* EIB202, assigning protein identities and quantifying protein abundance (Fig. 2).

### Effector candidates screened through comparative secretome

Quantitative and comparative secretome profiling on Δ*esaB* strain and Δ*esaB*Δ*esaN* strain identified ~300 differentially secreted proteins. Some of them share high values of Log_2_X. X denotes the ratio of specific protein abundance in ECPs of Δ*esaB* strain to that from the Δ*esaB*Δ*esaN* strain. The top 15 differently secreted proteins are listed in Table 1. Among them, four T3SS effectors (EseJ, EseG, EseH, and EseK) and three translocon components (EseB, EseC, and EseD) were previously reported (4 – 7, 16). Each of the substrates showed Log_2_X of >2, indicating that the screen is of high efficiency. Based on the secretion patterns exhibited by the known T3SS effectors (higher protein abundance in the culture supernatants of Δ*esaB* strain but low or no from that of the Δ*esaB*Δ*esaN* strain), two novel T3SS effector candidates were selected for further analysis on their secretion and translocation. One effector candidate encoded by the gene with the locus tag of ETAE_2009 is renamed as EseQ, and the other with the locus tag of ETAE_0559 is renamed as Trx2. In addition, several T6SS substrates (EvpM, EvpI, EvpP, and EvpQ) and Trxlp, an effector possibly secreted through type IV secretion system were also screened (31-34).

**TABLE 1:** A list of *E. piscicida* effectors identified from quantitative analyses of bacterial secretome^*a*^

Locus	Peptide (Δ*esaB*）	Peptide (Δ*esaB*Δ*esaN*)	Log_2_ X (protein abundance ratio)	Protein ID	Annotation	Secretion pathway	Reference
ETAE_2009	9	0	11	EseQ	Novel T3SS effector	T3SS	This study
ETAE_1757	10	0	11	EseH	T3SS effector	T3SS	6
ETAE_0888	53	0	9.4	EseJ	T3SS effector	T3SS	5
ETAE_0866	3	0	9.3	EseG	T3SS effector	T3SS	16
ETAE_1586	4	0	8.5	EseK	T3SS effector	T3SS	7
ETAE_0875	1	0	5.7	EsaP	T3SS early substrate	T3SS apparatus	4
ETAE_0559	1	0	3.5	Trx2	Novel T3SS effector	T3SS	This study
ETAE_0490	1	0	2.1	EseO	Effector	OMV	15
ETAE_2441	2	0	1.4	EvpM	T6SS protein	N/A	31
ETAE_2438	1	0	1.2	EvpJ	T6SS effector cargo protein	T6SS	31
ETAE_1309	1	0	1.1	ArtB	ArtB-like protein	Not determined	This study
ETAE_2428	2	0	0.6	EvpP	T6SS effector	T6SS	32
ETAE_2186	4	0	0.5	Trxlp	Thioredoxin (H-type, TRX-H)	T4SS, possible	33
ETAE_2437	15	5	0.5	EvpI	T6SS effector cargo protein	T6SS	31
ETAE_2037	12	3	0	EvpQ	Novel T6SS effector	T6SS	34

^*a*^ X denotes the specific peptide abundance in ECP of Δ*esaB* strain divided by that from Δ*esaB*Δ*esaN* strain; N/A: not available.

### Both EseQ and Trx2 are secreted and translocated in a T3SS-dependent manner

**Fig 3:**
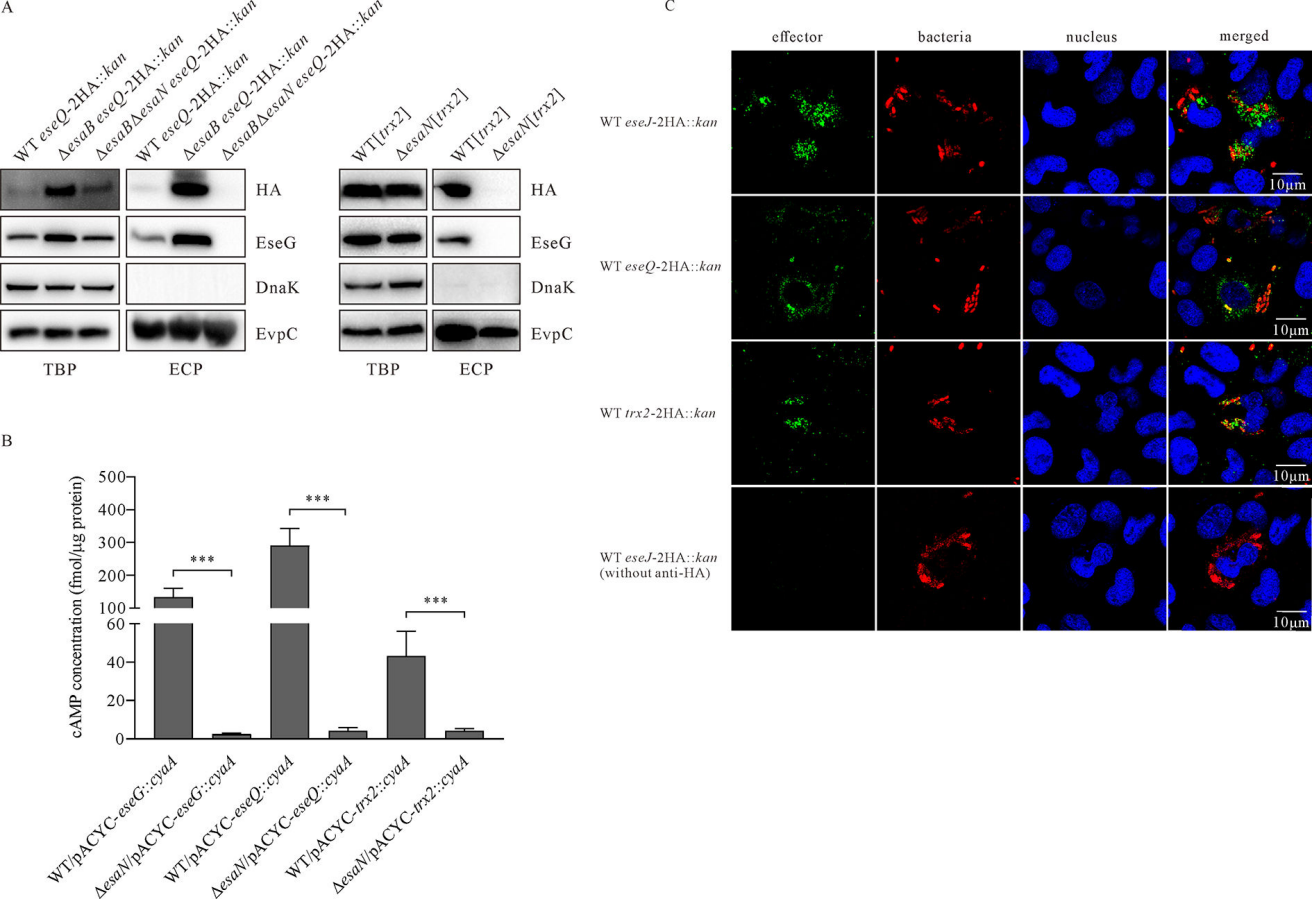
EseQ and Trx2 are novel T3SS effectors of *E. piscicida*. (**A)** EseQ and Trx2 are secreted into the culture supernatant in a T3SS-dependent manner. The immunoblotting for secretion of T3SS effector EseG and EseQ-2HA from *E. piscicida* wild-type strain, Δ*esaB eseQ-*2HA::*kan,* and Δ*esaB*Δ*esaN eseQ-*2HA::*kan* strain. EvpC, a protein secreted by T6SS, was included to indicate similar amount of protein loading per lane. DnaK, a bacterial cytosolic marker, was not detected in any ECPs, indicating that the detection of EseG and EseQ-2HA was not due to leakage from bacterial pellets. (**B)** EseQ and Trx2 are translocated in a T3SS-dependent manner. J774A.1 monolayers were infected with *E. piscicida* strains carrying the plasmid pACYC-*eseG::cyaA*, pACYC-*eseQ::cyaA,* or pACYC-*trx2::cyaA*. Intracellular cAMP levels were determined at 5 hpi, as described in Materials and Methods. Means ± SD from one representative experiment from three independent repeats are shown. ***, *P* < 0.001; NS, not significant. (**C)** Fluorescence microscopy images of the translocation of EseQ, Trx2, or EseJ by *E. piscicida* as revealed by immunofluorescent staining. EPC monolayers infected with *E. piscicida* strains were fixed at 5 hpi and stained with DAPI, anti-HA, anti-LPS, goat anti-rabbit IgG (Alexa 488), or goat anti-mouse IgG (Alexa 594).

To investigate the expression and secretion of EseQ, the chromosomal *eseQ* was labeled with 2HA tag. Highly increased EseQ-2HA was secreted from Δ*esaB eseQ*-2HA::*kan* strain than WT *eseQ*-2HA::*kan* strain, and no secretion from Δ*esaB*Δ*esaN eseQ*-2HA::*kan* strain was detected. Higher steady-state protein level of EseQ-2HA was detected from Δ*esaB* strain than from WT strain or Δ*esaB*Δ*esaN* strain. The expression and secretion pattern of EseQ are similar to that of T3SS effector EseG (Fig. 1A, left panel). EvpC was included to indicate similar level of protein loading per lane. DnaK was not detected in any ECPs, suggesting that the detected extracellular EseQ is not due to leakage from bacterial pellets. This indicates that EseQ can be secreted into culture supernatants in a T3SS-dependent manner under T3SS-inducing conditions. Trx2 is expressed by the genome at a low level and is hardly detectable (data not shown); hence, the pACYC-*trx2*-2HA was introduced into WT strain and Δ*esaN* strain to overexpress Trx2-2HA. In this way, Trx2 was found to be secreted in a T3SS-dependent manner (Fig. 3A, right panel). These results indicate that the secretion of either EseQ or Trx2 is dependent on T3SS.

Next, plasmids were constructed with *cyaA* fusion to the C-terminus of each candidate effector and introduced into the WT strain and the Δ*esaN* strain, respectively, before infecting the murine macrophage J774A.1 monolayers. The translocation of EseQ and Trx2 was investigated at 5 hours post infection (hpi). Infected J774A.1 monolayers were lysed, and the intracellular cAMP levels were measured as a read out of the translocation of effector candidates. As shown in Fig. 3B, the cAMP levels in cells infected with WT/pACYC-*eseG-cyaA* strain (positive control), WT/pACYC-*eseQ-cyaA* strain, and WT/pACYC-*trx*2-*cyaA* strain were 132.90 ± 27.56, 290.69 ± 52.31, and 43.20 ± 12.76 fmol per microgram of host protein, respectively; however, the cAMP levels in cells infected with Δ*esaN*/pACYC-*eseG-cyaA* strain (negative control)*,* Δ*esaN*/pACYC-*eseQ-cyaA* strain, and Δ*esaN*/pACYC-*trx*2-*cyaA* strain were 2.48 ± 0.61, 4.15 ± 1.79, and 4.22 ± 1.11 fmol per microgram of host protein, respectively. This indicates that both EseQ and Trx2 are translocated into host cells in a T3SS-dependent manner.

To confirm the delivery of EseQ and Trx2 into host cells, the translocation of EseQ and Trx2 was further examined by immunofluorescent staining. As shown in Fig. 3C, HA signaling was successfully detected in epithelioma papillosum of carp (EPC) cells when infected with WT *eseQ*-2HA::*kan* strain and WT *trx*2-2HA::*kan* strain, which are similar to the positive control, EPC monolayers infected with WT *eseJ*-2HA::*kan* strain. When the anti-HA antibody was omitted from immunofluorescent staining, no HA signal was detected from EPC cells infected with the WT *eseJ*-2HA::*kan* strain. This indicates the specificity of the detection and the translocation of EseQ and Trx2, thus demonstrating that EseQ and Trx2 are novel T3SS effectors of *E. piscicida*.

### Trx2 suppresses macrophage apoptosis

**Fig 4:**
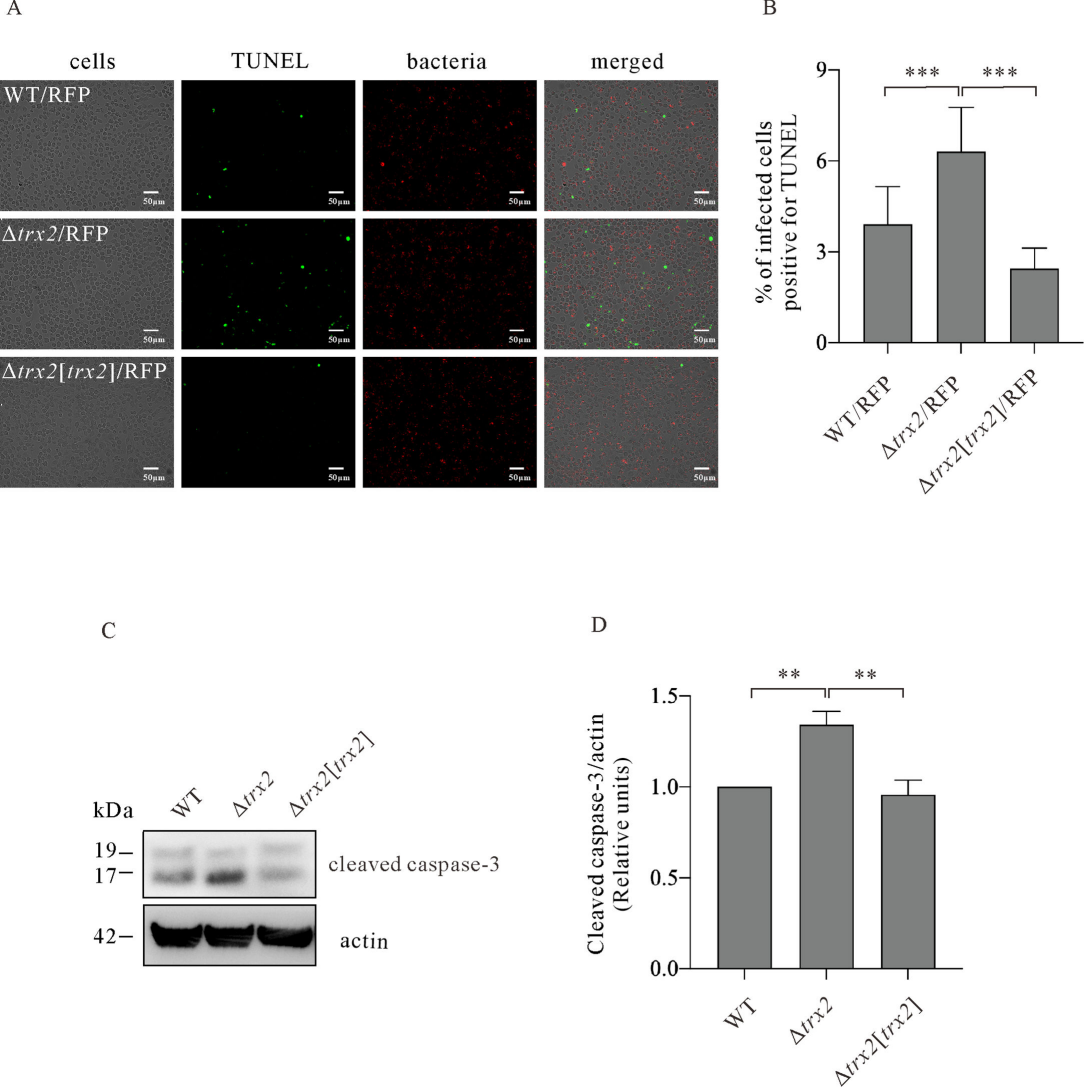
Trx2 suppresses macrophage apoptosis (**A)** Confocal laser scanning micrograph of TUNEL-stained J774A.1 cells infected with *E. piscicida* strains. J774A.1 cells were infected with wild-type (WT)/RFP strain, Δ*trx2*/RFP strain, and Δ*trx2* (*trx2*)/RFP strain at MOI 10, and at 2 hpi, the fragmentation of nuclear DNA from infected J774A.1 cells was examined by TUNEL staining. (**B)** Percentage of infected J774A.1 cells was positive for TUNEL. TUNEL-positive cells from more than 500 cells per view were counted. The graph shows the relative ratio (means ± SD) of one representative experiment from three independent repeats. ***, *P* < 0.001. (**C)** The immunoblotting on cleaved caspase-3 from J774A.1 cells infected with WT strain, Δ*trx2* strain, and Δ*trx2* [*trx2*] strain. Actin was used to indicate that similar amounts of protein were loaded per lane. The experiment was repeated independently for four times, and one representative image is shown. (**D)** Quantitative analysis of cleaved caspase-3 from J774A.1 cells infected with *E. piscicida* strains indicated in Fig. 4C. The levels of cleaved caspase-3 were quantified by densitometry and normalized against those of actin. The graph shows the relative ratio (means ± SEM) of cleaved caspase-3 from four independent experiments. **, *P* < 0.01.

Mammalian Trx2 associates with mitochondrial ASK1 and inhibits the JNK-independent apoptosis (35). Does *E. piscicida* T3SS effector Trx2 play a role in host apoptosis? To investigate, J774A.1 monolayers were infected with WT/RFP, Δ*trx2*/RFP, and Δ*trx2*[*trx2*]/RFP at MOI 10. Terminal deoxynucleotidyl transferase dUTP nick-end labeling (TUNEL) assay was performed at 2 hpi to probe for fragmented DNA, which is a hallmark of apoptosis (Fig. 4A). It was found that 6.31 ± 1.46% of Δ*trx2*/RFP strain-infected J774A.1 cells were TUNEL positive; however, only a small amount of TUNEL-positive cells was detected from WT/RFP-infected (3.91 ± 1.24%) and Δ*trx2*[*trx2*]/RFP-infected (2.45 ± 0.67%) J774A.1 cells (Fig. 4B). Caspase-3 cleavage is another hallmark of apoptosis. The protein levels of cleaved caspase-3 from J774A.1 macrophages infected with different *E. piscicida* strains were examined by immunoblotting. A significantly increased protein level of cleaved caspase-3 was detected from J774A.1 cells infected with the Δ*trx2* strain at 3 hpi, as compared with that of WT or Δ*trx2*(*trx2*) strain (Fig. 4C and D). Taken together, T3SS effector Trx2 inhibits apoptosis of J774A.1 cells which are infected with *E. piscicida*.

### Trx2 suppresses the activation of NF-κB pathway

**Fig 5:**
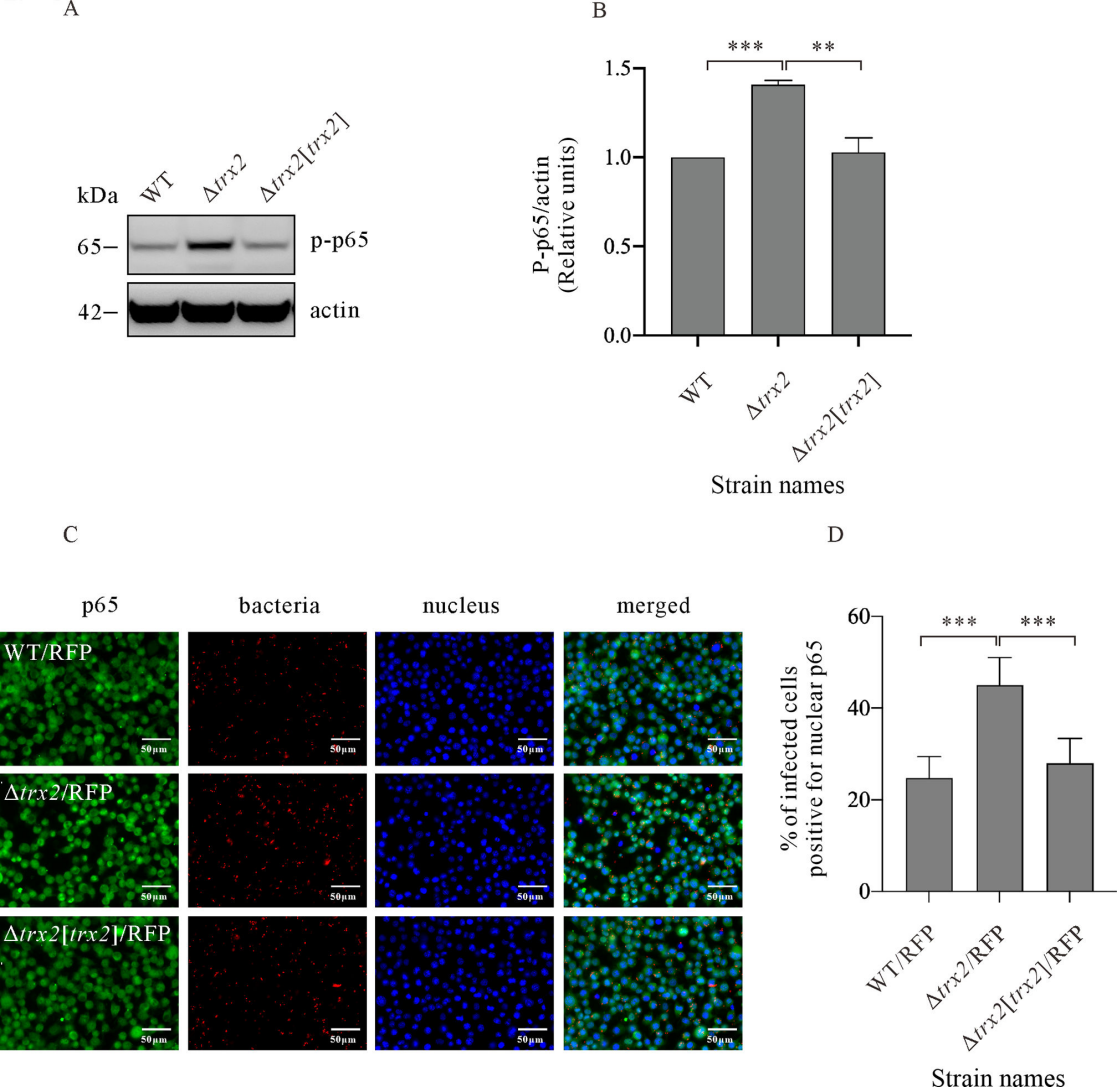
Trx2 inhibits the phosphorylation of p65 and its translocation into nucleus. (**A)** Immunoblotting on p-p65 (ser 536) from J774A.1 cells infected with wild-type (WT) strain, Δ*trx2* strain, and Δ*trx2*[*trx2*] strain. Actin was used to indicate that similar amounts of protein were loaded per lane. The experiment was repeated independently for three times, and one representative image is shown. (**B)** Quantitative analysis of p-p65 from J774A.1 cells infected with *E. piscicida* strains indicated in Fig. 5A. The level of p-p65 was quantified by densitometry and normalized against that of actin. The graph shows the relative ratio (means ± SEM) of p-p65 from three independent experiments. **, *P* < 0.01; ***, *P* < 0.001. (**C)** J774A.1 cells infected with *E. piscicida* strains were immunofluorescently stained with p65 antibody (green) to monitor nucleus translocation of p65. *E. piscicida* were indicated by RFP (red) and nucleus by DAPI (blue). Scale bar represents 50 µm. (**D)** Statistical analysis on the percentage of infected cells was positive for nucleus p65 of Fig. 5C. Means ± SD values for a representative experiment from three times independent repeat are shown. ***, *P* < 0.001.

*E. piscicida* effector protein Trxlp inhibits the nuclear translocation of NF-κB (36). Trx2 shares 53% similarity with Trxlp. Is Trx2 involved in NF-κB pathway? To investigate, the protein levels of phosphorylated p65 (p-p65) in J774A.1 macrophages infected with WT/RFP, Δ*trx2*/RFP, and Δ*trx2*[*trx2*]/RFP strains were examined. Significantly increased steady-state protein levels of p-p65 were detected in J774A.1 cells infected with Δ*trx2* strain as compared with that infected with WT or Δ*trx2*(*trx2*) strain (Fig. 5A and B). Activation of NF-κB signaling pathway is followed by nuclear translocation of NF-κB dimers (p65/p50), resulting in a rapid and transient transcriptional activation of proinflammatory cytokine (37). Does Trx2 inhibit nuclear translocation of p65? For investigation, p65 present in the cytoplasm and nucleus of J774A.1 cells were examined by immunofluorescence staining. It was observed that depletion of Trx2 resulted in a significant increase of p65 present in the nucleus of J774A.1 cells (Fig. 5C and D). Taken together, we have shown that Trx2 suppresses p65 phosphorylation, inhibits translocation of p65 into the nucleus, and thereby blocking NF-κB pathway.

### Trx2 slightly but significantly contributes to the pathogenesis of *E. piscicida* in fish

**Fig 6:**
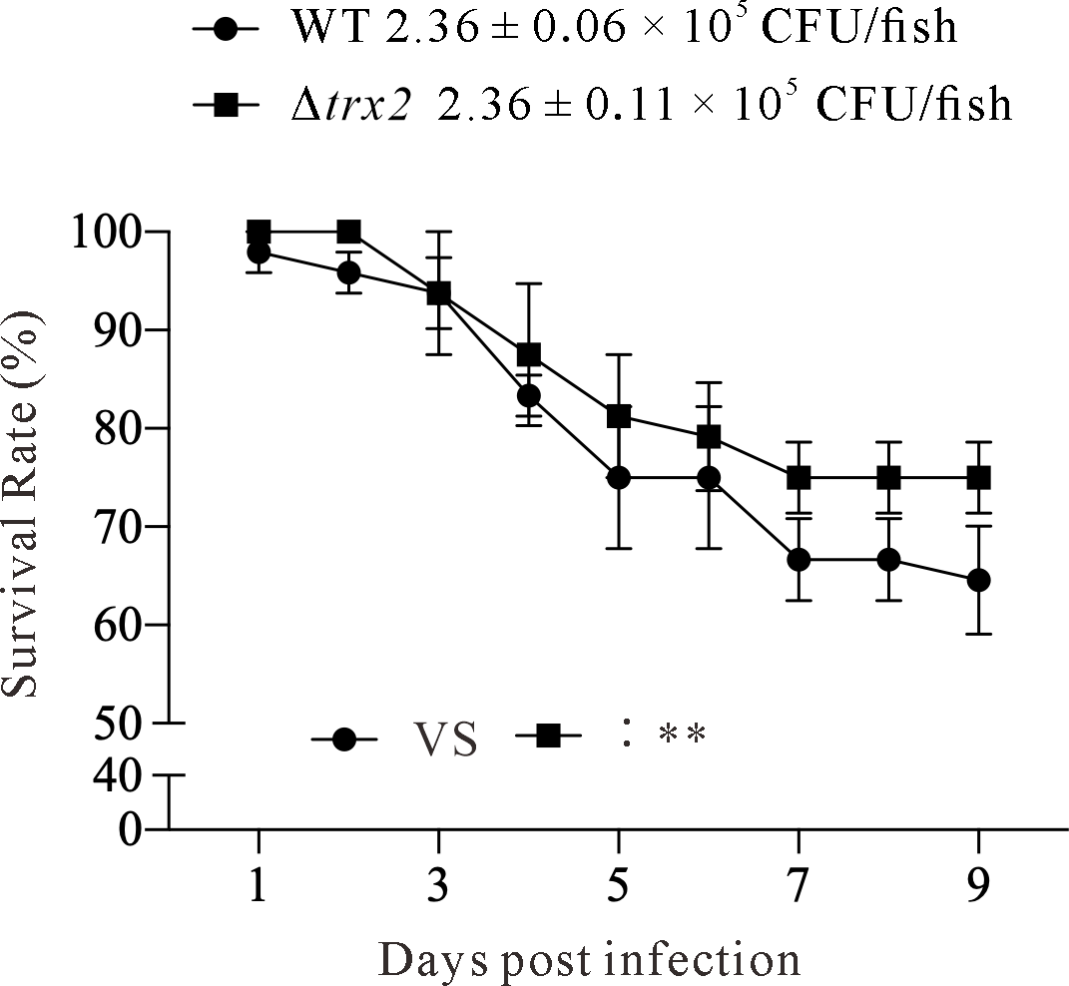
Trx2 slightly but significantly contributes to the virulence of *E. piscicida* in blue gourami. Sixteen naïve blue gourami fish were injected intramuscularly with wild-type (WT) strain and Δ*trx2* strain. Survival rates of blue gourami fish were monitored for 9 days. Means ± SEM from three independent experiments are shown. **, *P* < 0.01.

To learn the contribution of Trx2 to *E. piscicida* virulence, the survival rates of blue gourami infected with *E. piscicida* wild-type strain (WT) and the isogenic Δ*trx2* strain were compared. The naïve blue gourami were infected by intramuscular injection nearby dorsal fin with dosage of (2.36 ± 0.06) × 10^5^ CFU of WT strain or (2.36 ± 0.11) × 10^5^ CFU of Δ*trx2* strain. The survival rates were monitored for 9 days. From three independent experiments, we demonstrated that infection with the Δ*trx2* strain exhibited a slightly increased accumulated survival rates when comparing to that of WT strain, and the difference between the two groups is significantly different as revealed by the paired *t* test, which was used to calculate the two-tailed *P-*value (Fig. 6). This indicates that Trx2 slightly but significantly contributes to the virulence of *E. piscicida*.

## DISCUSSION

Depletion of EsaB disrupts the T3SS gatekeeper-containing protein complex EsaB-EsaL-EsaM, stimulating the secretion of T3SS effectors in *E. piscicida*. In this study, quantitative and comparative proteomics at the secretome of *E. piscicida* Δ*esaB* strain and Δ*esaB*Δ*esaN* strain (T3SS mutant), together with CyaA-based translocation assay revealed that EseQ and Trx2 are the novel T3SS effectors of *E. piscicida*. In addition, Trx2 has been revealed to suppress apoptosis and inhibit NF-κB pathway, thus contributing to the pathogenesis of *E. piscicida*.

*Salmonella* T3SS gatekeeper protein SsaL inhibits the secretion of SPI-2 effectors at acidic pH (38, 39). EsaL of *Edwardsiella* is a functional ortholog of SsaL of *Salmonella* and SepL of enterohemorrhagic *E. coli* (28). The *sepL* mutant strains of pathogenic *Escherichia coli* highly secrete T3SS effectors and their secretomes were subjected to proteomic analysis to search for novel T3SS effector candidates (40 – 42). However, similar level of the T3SS effector EseJ was secreted by *esaL* mutant and *E. piscicida* wild-type strain (28). EsaB is the homolog of SpiC, and the depletion of EsaB disrupts the T3SS gatekeeper-containing protein complex EsaB/EsaL/EsaM, resulting in greatly increased secretion of T3SS effector EseJ when cultured in DMEM (28). Therefore, the secretomes of Δ*esaB* strain and Δ*esaB*Δ*esaN* strain (T3SS mutant) were compared to search for novel T3SS effector candidates in *E. piscicida*.

By the method we established in this study, ~300 proteins were identified from the secretome of either Δ*esaB* strain or Δ*esaB*Δ*esaN* strain; in contrast, only ~30 proteins were identified when the secretomes concentrated by centrifugal filter devices were subjected to trypsin digestion directly. It is speculated that ECPs denature before loading into SDS-PAGE gel block secretome from degradation by *E. piscicida*-secreted protease(s), also brief electrophoresis of the SDS-PAGE gel helps to remove low molecular weight impurities from existing DMEM. It is speculated that denaturation of the secretome along with short electrophoresis contributes to the efficiency of trypsin digestion; hence, more proteins from the *E. piscicida* secretome have been identified.

Trx2 (ETAE_0559) shares 70.42% identity to thioredoxin 2 from *Yersinia pestis*, according to the Swiss-model analysis (https://swissmodel.expasy.org/). Host cell thioredoxin 2 inhibits the apoptosis mediated by mitochondria-located ASK1 in a Jnk-independent manner (35). Also, the *E. piscicida* thioredoxin-like effector Trxlp inhibits ASK1-MAPKs signaling and nuclear translocation of NF-κB to promote pathogenesis. Trxlp mimics endogenous thioredoxin to abrogate ASK1 homophilic interaction and phosphorylation, thereby suppressing the phosphorylation of Erk1/2- and p38-MAPK downstream signaling cascades, and it can also inhibit cellular redox signaling and nuclear translocation of NF-κB, thereby facilitating survival and replication (33, 36). *E. piscicida* Trx2, which shares 53% similarity to Trxlp, has been revealed to inhibit p65 nuclear translocation. It is speculated that Trx2 inhibits NF-κB pathway probably through interaction with ASK1 in host cells. The interaction of ASK1 and *E. piscicida* Trx2 translocated awaits further study.

A novel T6SS effector EvpQ encoded by the locus tag of ETAE_2037 was screened using the method established in this study (34). The protein abundance of EvpQ is of the same as Log_2_X equals 0, although 12 kinds of peptides and 3 kinds of peptides of EvpQ were identified from the ECPs of Δ*esaB* strain and Δ*esaB*Δ*esaN* strain, respectively (Table 1). This suggests that secretion of EvpQ is not dependent on T3SS (Table 1). EvpP is also a T6SS effector of *E. piscicida* (32); however, secretion of EvpP partially depends on T3SS as shown in Table 1. Indeed, EvpP is secreted partially in a T3SS-dependent manner, this was repeatedly demonstrated by immunoblotting (data not shown). It is speculated that more novel T6SS effector candidates will be unraveled in future based on their similar secretion level of the specific protein from the Δ*esaB* strain and Δ*esaB*Δ*esaN* strain.

*E. piscicida* PPD130/91, previously named *E. tarda* PPD130/91, was a fish isolate (43). It replicates robustly in fish macrophage, murine bone marrow-derived macrophage or murine macrophage cell lines J774A.1 and Raw264.7 (4, 44, 45). The replication of *E. piscicida* in either fish or murine macrophage depends on an active T3SS, which is regulated by temperature (4, 16, 46). PhoQ-PhoP is one of the two-component systems of *E. piscicida*, PhoQ responds to temperature change. Activation of the PhoQ histidine kinase over the temperature range from 23°C to 35°C leads to autophosphorylation, and the PhoP binds directly to the PhoP box within the promoter region of *esrB* to activate its transcription; the EsrB protein then integrates the signal from another two-component system EsrA-EsrB, activating the transcription of T3SS genes (46). Secretion of T3SS effectors decreases at 37°C as compared to 35°C (16). To maximize the expression of *E. piscicida* T3SS effectors, murine macrophage J774A.1 were infected at 35°C in this study, whereas the blue gourami fish were infected at 28°C, at which temperature fish are cultured.

In conclusion, we used quantitative and comparative proteomic profiling to screen candidate effector that is highly secreted by Δ*esaB* strain but are not, or to a lesser extent, secreted by the Δ*esaB*Δ*esaN* strain. This prompted us to present EseQ and Trx2 as novel T3SS effectors of *E. piscicida*. Trx2 inhibits apoptosis, suppresses NK-κB pathway, and contributes to virulence of *E. piscicida* in fish infection model. Future research will be focused on elucidating the physiological function of the newly identified effectors to better understand the pathogenesis of *E. piscicida*.

## MATERIALS AND METHODS

### Bacterial strains, cell, and growth conditions

Bacterial strains and plasmids used in this study are described in Table 2. *E. piscicida* PPD130/91 (43) and its derived strains were grown in tryptic soy broth (TSB; BD Biosciences) at 28°C, and *Escherichia coli* strains were cultured in Luria-Bertani broth (LB; BD Biosciences). *E. piscicida* strains were grown in DMEM (Thermo Fisher) at 25°C under a 5% (vol/vol) CO_2_ atmosphere to switch on its T3SS. When required, appropriate antibiotics were supplemented at the following concentrations: 100 µg/mL ampicillin (Sigma), 50 µg/mL kanamycin (Sigma), 12.5 µg/mL colistin (Sigma), 15 µg/mL tetracycline (Amresco), and 34 µg/mL chloramphenicol (Amresco).

**TABLE 2:** Strains and plasmids used in this study^*a*^

Strain or plasmid	Description and/or genotype	Source
Bacteria		
*E. piscicida*		
*E. piscicida* PPD130/91	Wild type, Km^s^, Col^r^, Amp^s^, LD_50_ = 10^5.0^	43
Δ*esaB*	PPD130/91, *esaB* in-frame deletion of aa 1 to 149	28
Δ*esaN*	PPD130/91, *esaN* in-frame deletion of aa 21 to 421	16
Δ*esaB*Δ*esaN*	PPD130/91, in-frame deletion of *esaB* and *esaN*	This study
WT/pACYC-*eseG*::*cyaA*	PPD130/91 with pACYC-*eseG*::*cyaA*	47
WT/pACYC-*eseQ*::*cyaA*	PPD130/91 with pACYC-*eseQ*::*cyaA*	This study
WT/pACYC-*trx2*::*cyaA*	PPD130/91 with pACYC-*trx2*::*cyaA*	This study
Δ*esaN*/pACYC*-eseG*::*cyaA*	Δ*esaN* with pACYC-*eseG*::*cyaA*	47
Δ*esaN*/pACYC*-eseQ*::*cyaA*	Δ*esaN* with pACYC-*eseQ*::*cyaA*	This study
Δ*esaN*/pACYC*-trx2*::*cyaA*	Δ*esaN* with pACYC-*trx2*::*cyaA*	This study
WT *eseQ*-2HA::*kan*	PPD130/91 with chromosomal expression of *eseQ*-2HA, Km^r^	This study
Δ*esaB*Δ*esaN eseQ*-2HA::*kan*	Δ*esaB*Δ*esaN* with chromosomal expression of *eseQ*-2HA, Km^r^	This study
Δ*esaB eseQ*-2HA::*kan*	Δ*esaB* with chromosomal expression of *eseQ*-2HA, Km^r^	This study
WT *trx2*-2HA::*kan*	PPD130/91 with chromosomal expression of *trx2*-2HA, Km^r^	This study
WT *eseJ*-2HA::*kan*	PPD130/91 with chromosomal expression of *eseJ*-2HA, Km^r^	This study
WT[*trx2*]	PPD130/91 with pACYC-*trx2*-2HA	This study
Δ*esaN*[*trx2*]	Δ*esaN* with pACYC-*trx2*-2HA	This study
Δ*trx2*	PPD130/91, *trx2* in-frame deletion of aa 1 to 144	This study
Δ*trx2*[*trx2*]	Δ*trx2* with pACYC-*trx2*-2HA	This study
WT/RFP	PPD130/91 with pFPV25.1-RFP	9
Δ*trx2*/RFP	Δ*trx2* with pFPV25.1-RFP	This study
Δ*trx2*[*trx2*]/RFP	Δ*trx2* with pACYC-*trx2*-2HA and pFPV25.1-RFP	This study
*E. coli*		
DH5α	α Complementation	Stratagene
S17-1 λpir	RK2 *tra* regulon, λ*pir*	48
Plasmids		
pFPV25.1-*rfp*	Derivative of pBR322 with *rfp* under the control of the constitutive promoter, expressing red fluorescent protein	9
pRE112	Suicide plasmid, *pir* dependent, Cm^r^, *oriT*, *oriV*, *sacB*	49
pACYC-*escE*::*cyaA*	Tet^r^, Cm^r^	47
pKD46	Red helper plasmid, Amp^r^	50
pKD4	Template plasmid for PCR amplifies FRT-flanked resistance gene, Km^r^	50
pSU315	Template plasmid with FLP recognition target site and 2 HA tag sequence, Amp^r^, Km^r^	51

^*a*^ Col, colistin; Amp, ampicillin; Tet, tetracycline; Cm, chloramphenicol. Superscripts: r, resistance; s, sensitivity.

Murine J774A.1 macrophages were cultured at 35°C in DMEM (Gibco) with 10% fetal bovine serum (FBS). Epithelioma papillosum cyprini (EPC) cells (52) were grown at 25°C in MEM medium (HyClone) supplemented with 10% FBS.

### Mutants and plasmids construction

Non-polar deletion mutants of *trx*2 were generated by *sacB*-based allelic exchange (49) as described previously (53). Briefly, two PCR fragments were generated from PPD130/91 genomic DNA with the primer pairs *trx*2-for plus *trx*2-int-rev and *trx*2-int-for plus *trx*2-rev. The resulting products were a 1027-bp fragment containing the upstream region of *trx*2 and a 1062-bp fragment containing the downstream region of *trx*2. A 15-bp overlap in the sequences permitted their fusion by a second round of PCR using the *trx*2-for and *trx*2-rev primers. The resulting PCR product, with the deletion of the 1–144 aa of Trx2, was digested and ligated into the KpnI restriction site of the pRE112 suicide vector (49) to create pRE-Δ*trx*2, which was transferred into *E. coli* strain S17-1 λpir (48) to conjugate with *E. piscicida* PPD130/91. Deletion mutant strains were screened on 10% sucrose-tryptic soy agar (TSA) plates and were verified by PCR and sequencing. None of the mutant strains obtained show growth defect when cultured in either TSB or DMEM. Based on Δ*esaB* strain, Δ*esaB*Δ*esaN* strain was screened according to the ECP profiles. All the primers used are listed in Table 3.

**TABLE 3:** Oligonucleotides used in this study

Designation	Nucleotide sequence
*trx2-for*	CTCGATATCGCATGCGGTACCCGGAAGGTATCCAGCGCC
*trx2*-int-rev	GATTATCCTCAGACATAAAACGGCG
*trx2*-int-for	TGTCTGAGGATAATCTCCCAATATCAACGGCCCAT
*trx2*-rev	CAAGCTTCTTCTTCTAGAGGTACCGATAGCCAAATCCGGGACACA
*eseQ*::*cyaA*-for	GAGGATCCCGCTACCAAATAAGCAATGTG
*eseQ*::*cyaA*-rev	GGAGATCTATGTTCCGCGAGCCACCGCTC
*trx2*::*cyaA*-for	CCACACCCGTCCTGTGGATCCGCGAAGATTACCTGATGTGAGTACG
*trx2*::*cyaA*-rev	ACCTCCGCCAGATCTCTCGAGGGCCTGCTTGGCCAACTG
*eseQ*-2HA-for	ATTAGCACTGGTCAGAGAGAAAGCGGAGCGGTGGCTCGCGGAACATTATCCGTATGATG TGCCGGACTATGCGTATCCGTATGATGTTCCTGAT
*eseQ*-2HA-rev	TGATTGCGTAATCGTCGTGGGTATAACCCTCTTAAAAAGCCATATGAATATCCTCCTTAGT
*trx2*-2HA-for	ACCGTTTGAAGCCTGGCTGGATGAGCAGTTGGCCAAGCAGGCCTATCCGTATGATGTG CCGGACTATGCGTATCCGTATGATGTTCCTGAT
*trx2*-2HA-rev	TAAACAAGCCTCGTCACCGGGTACAGCGTCAAACGGCCCCGCGCGCATGGCATATGAAT ATCCTCCTTAGT
*eseJ*-2HA-for	GCACAGCCGCCTGAACGGGCATGAGACGGCGGCGGCGATATATCCGTATGATGTTCCT GATTATGCTAGCCTCTAGTAATGTAGGCTG
*eseJ*-2HA-rev	ATCGTCACGGTGCTACGCCGCCTGACGCGGCGGCGTAAATCCCATATGAATATCCTCCTTAG
pACYC-*trx2*-com-for	ATGAATGCTCATCCGGAATTCAGCTGCTATCTATTATAGTCATTGGGTAA
pACYC-*trx2*-com-rev	CCTGCCACTCATCGCAGTACTTACTAGAGGCTAGCATAATCAGGAACATCATACGGATAGGC CTGCTTGGCCAACTG

The DNA sequence including the *trx2* gene and its ribosome binding site was amplified with primers *trx2*-com-for and *trx2*-com-rev and ligated into the EcoRI and ScaI restriction sites of pACYC-184 (Amershan) to create pACYC-*trx2*-2HA. The *eseQ* gene without its stop codon was amplified with primers *eseQ-cyaA*-for and *eseQ-cyaA*-rev and digested with KpnI and BglII to replace *escE* from pACYC-*escE*::*cyaA* (47), yielding pACYC-*eseQ*::*cyaA*. Similarly, pACYC-*trx2*::*cyaA* was constructed. The plasmids constructed were verified by DNA sequencing and introduced into both *E. piscicida* PPD130/91 wild-type strain and the Δ*esaN* strain by electroporation.

### Epitope tagging of the chromosomal copy of effector candidates with 2HA tag

To tag the chromosomal copy of *eseQ* with 2HA epitope, the λ Red recombination system was used as previously described (50, 51), with slight modification. Briefly, with pSU315 as the template, forward primers containing the C-terminal sequence (without the stop codon) of *eseQ* and the reverse primer corresponding to a chromosomal region downstream of *eseQ* were used to amplify the kanamycin resistance gene (*kan*). The PCR product was electroporated into competent cell of *E. piscicida* PPD130/91 transformed with pKD46 (50). By inducing with L-arabinose at 30°C, WT *eseQ*-2HA::*kan* strain was screened on TSA-Km plates and verified by immunoblotting with anti-HA antibody and sequencing. Similarly, the chromosomal copy of *trx*2 and *eseJ* was tagged and verified, respectively.

### Secretome preparation

Three colonies from fresh plates were inoculated into 5 mL of DMEM medium and cultured for 24 hours before being subcultured at 1:200 into 30 mL of DMEM and cultured at 25°C under a 5% (vol/vol) CO_2_ atmosphere for 24 hours. Culture supernatants (extracellular proteins, ECPs) were filtered with a 0.22-µm filter before being concentrated with Amicon Ultra-15 centrifugal filter devices with 3 kDa molecular weight cut-off (Millipore). The culture supernatants were concentrated to 200 µL per strain.

### Mass spectrometry and data analysis

Concentrated culture supernatants were pre-purified by loading into SDS-PAGE gel for a short electrophoresis. The protein bands were cut and subjected to in-gel digestion with trypsin prior to mass spectrometric analysis. Briefly, gel slices were minced and destained with 30% ACN/100 mM NH_4_HCO_3_ before drying in a vacuum centrifuge. The in-gel proteins were reduced with dithiothreitol (10 mM DTT/100 mM NH_4_HCO_3_) for 30 minutes at 56°C, then alkylated with iodoacetamide (200 mM IAA/100 mM NH_4_HCO_3_) in the dark at room temperature for 30 minutes. Gel slices were then briefly rinsed with 100 mM NH_4_HCO_3_ and ACN, respectively, before proteins in gel being digested overnight in 12.5 ng/µL trypsin in 25 mM NH_4_HCO_3_ at 37°C for 20 hours. The peptides were extracted three times with 60% ACN/0.1% TFA. The organic solvents from all extracts were removed by vacuum centrifugation. After desalination, the enzymatic hydrolysate was freeze dried, re-dissolved in 0.1% FA solution, and stored at −20°C.

Mass spectrometry and preliminary data analysis were performed by Shanghai Applied Protein Technology. The QE mass spectrometer is used in this experiment. Each sample was injected for nano-LC-MS/MS analysis. The mass-to-charge ratios of peptides were collected according to the following method: 20 fragment profiles (MS2 Scan) were collected after complete scan. The original mass spectrometry documents (raw file) were acquired, and Mascot2.2 software was used to search the *E. piscicida* EIB202 genome database to identify proteins from the secretomes.

### Immunoblotting

The total bacterial pellets or the ECPs of *E. piscicida* strains were separated by SDS-PAGE gel and transferred to polyvinylidene difluoride membranes (0.45 µm or 0.22 µm). Immunoblotting analyses were carried out with primary antibodies specific for HA at a dilution of 1:3,000 (Sigma), rabbit anti-EseG at 1: 2,000 (16), rabbit anti-EseJ at 1: 2,000 (5), rabbit anti-EvpC at 1: 5,000 (31), rabbit anti-DnaK at 1:10,000 (Cusabio Technology LLC), and the secondary antibody of horseradish peroxidase-conjugated goat anti-mouse IgG at 1:2,500 (Millipore) or goat anti-rabbit IgG at 1:2,500 (Millipore). Antigen-antibody complexes were visualized with Super Signal West Pico Chemiluminescent Substrate (Thermo), followed by exposure using a ChemiDoc MP imaging system (Bio-Rad). Immunoblotting experiments were independently repeated at least three times.

J774A.1 monolayers were infected at 35°C as described previously (9). At 3 hpi, the cell culture supernatants were collected, and the cells were lysed with immunoprecipitation lysis buffer for 10 minutes on ice. The supernatants together with the cell lysates were precipitated by methanol and chloroform as described by Wang et al. (54). The pellets obtained were dried and dissolved in sodium dodecyl sulfate loading buffer. The protein samples were subjected to NuPAGE 10% bis-Tris gel (Novex) electrophoresis for immunoblotting analyses and probed overnight at 4°C with rabbit anti-cleaved caspase-3 (Asp175) at a 1:1,000 dilution, rabbit anti-phosphorylated p65 monoclonal antibody (Ser 536, CST) at a 1:1,000 dilution, and rabbit anti-actin polyclonal antibody (ABclonal) at a 1:5,000 dilution.

### Apoptosis assay by *in situ* detection of fragmented DNA

For microscopic analysis of apoptosis, cells were seeded 24 hours prior to infection in 24-well plates with coverslips as previously described (9). At 2 hours post infection, the infected monolayers were rinsed with sterile phosphate buffered saline (PBS) and fixed with 4% paraformaldehyde (PFA). Next, the fixed monolayers were permeabilized with 0.2% Triton X-100, levels of apoptosis in infected J774A.1 cells were measured by the TUNEL assay using the DeadEnd Fluorometric TUNEL System (Promega) according to the manufacturer’s instructions. Images were taken using a Leica fluorescence microscope and scored for the number of TUNEL-positive cells/positively infected cells.

### CyaA-based translocation assay

CyaA-based translocation assay was performed as previously described (5). Briefly, 5 hours post infection, the J774A.1 cell monolayers were washed twice with PBS before being lysed with sample diluent [supplied with the cyclic AMP (cAMP) immunoassay kit, supplemented with 0.2% Triton X-100], and the cAMP levels were evaluated using a cAMP enzyme immunoassay system (Arbor Assays). The translocation efficiency was calculated from the mean of three wells per infection, and the experiment was repeated three times independently.

### Immunofluorescence staining

The immunofluorescence staining was performed as previously described (55). Briefly, the EPC monolayers were infected with WT *eseQ*-2HA::*kan* strain, WT *trx2*-2HA::*kan* strain, and WT *eseJ*-2HA::*kan* strain at MOI 10. At 5 hpi, the EPC monolayers were fixed in 4% PFA and stained with rabbit anti-HA (Cell Signaling Technology) at 1:200, mouse anti-LPS at 1:200 (56), goat anti-rabbit IgG (Alexa 488; Molecular Probes) at 1:200, and goat anti-mouse IgG (Alexa 594; Molecular Probes) at 1:200 and sealed with anti-fading mounting reagent with DAPI before images were photographed under a confocal microscope (NOL-LSM 710; Carl Zeiss).

To determine the nucleus accumulation of p65, J774A.1 cells were infected with WT/RFP, Δ*trx2*/RFP, and Δ*trx2*[*trx2*]/RFP strains at MOI 10 for 2 hours. After fixation, the monolayers were stained with mouse anti-p65 (Cell Signaling Technology) at 1:200, and goat anti-mouse IgG (Alexa 594; Molecular Probes) at 1:200 and sealed with anti-fading mounting reagent with DAPI before images were photographed under a confocal microscope (NOL-LSM 710; Carl Zeiss).

### Single-strain infection in blue gourami fish

Blue gourami infection was performed according to the Guide for the Care and Use of Laboratory Animals of the Institute of Hydrobiology, Chinese Academy of Sciences. *E. piscicida* wild-type strain and Δ*trx2* strain were subcultured in TSB to exponential growth phase (OD_540_, ~0.5). Sixteen healthy blue gourami fish (7.63 ± 1.48 g) were used per infection. Each blue gourami fish was injected intramuscularly near the dorsal fin with (2.36 ± 0.06) × 10^5^ CFU of *E. piscicida* wild-type strain, or (2.36 ± 0.11) × 10^5^ CFU of Δ*trx2* strain. Fish were maintained at 28°C, and mortality was recorded for 9 days.

### Statistical analysis

All data are presented as means ± SEM or means ± SD. Statistical tests were applied to data from at least three independent experiments or one representative experiment. Probability (*P*) values were calculated by Student’s *t* test for Fig. 3B; Fig. 4B and D and Fig. 5B and D.

The paired *t* test was used to calculate the *P-*value of survival rates between WT-infected and the ∆*trx2-*infected blue gourami. A *P*-value of < 0.05 was considered significant.
